# Hospital and Institutional News

**Published:** 1920-11-13

**Authors:** 


### HOSPITAL AND INSTITUTIONAL NEWS.

### TO HONOUR THE UNKNOWN.

At the very moment these pages go to press the very beautiful ceremonies are closing by which the nation, in giving the highest honour to one unknown soldier from the thousands of nameless battlefield graves, is paying a unique tribute to the many who sacrificed their lives for England—but vanished utterly in the giving. So much has already been written upon the true meaning of this particular commemoration of the great Armistice that it is here redundant to repeat either the details or their significance. But to the hospital world and its many workers the years of war showed so many diverse glimpses of sorrow and anguish at home, as well as in the actual fighting areas, that there must be many of our readers who will welcome and appreciate with exceptional keenness this latest sequence to a hard-won victory. The chosen events show a touch of that high imagination which does not often blend itself into our more recent public life. Too many of our rushed-through ceremonies have done but scant justice to facts as they are. But this is a great plan, and worthy of a great nation. There is about it that simple dignity, a return of that type of thought which belonged to other and more spacious days when men had more time and the will to consider and create in the light of far higher ideals. One thought is, however, insistent at this moment. We are doing much to honour the dead. Do we not sometimes tend to forget the living? The wounded—now, perhaps, we should say the disabled—are still with us in plenty. Official care they receive; but do the public, the providers of entertainment, subscriptions, etc., remember them equally well? It is just and right to honour the dead, but it is good also to comfort the living, who, though not making the supreme sacrifice, have in many cases suffered the more.

### THE MINISTRY OF HEALTH BILL.

The second reading of the Ministry of Health (Miscellaneous Provisions) Bill was commenced on November 4, continued on November 9 before a very thin House, and carried. The hospital clauses, which are most interesting to our readers, came forward in pretty much the same form as in the original draft. There were, however, one or two alterations which are of some importance, although they do not dispel the criticism of the rather weighty opposition inside as well as outside the House of Commons. Clause 11 has been amended to enable the Ministry of Health to keep a tighter hold over local authorities in respect of the foundation of municipal hospitals. Authorities will be able to take over Poor-law institutions, but the provision for building has been abundant; this power shall not extend to taking over voluntary hospitals, but allows the authorities to make voluntary contributions. Dr. Addison would not express an opinion as to whether Bradford and Willesden have exceeded powers under the Public Health Act when they proceeded with the establishment of municipal hospitals, but he supplied the interesting information that under private Bills the city of Liverpool has taken power to contribute £5,000 a year to the Royal Infirmary, and at Salford a rate has been made limited to a penny in the pound, the produce of which is to be handed to voluntary hospitals. Neither in Dr. Addison's speeches nor in the debate that followed (both largely referring to other parts of the measure) did anything arise which alters materially the position of voluntary hospitals in respect of the Bill. The Minister made certain references to the financial position of voluntary hospitals and allusion to these will be found in this week's leading article.

### FEDERATION OF MEDICAL AND ALLIED SOCIETIES.

The conference of representatives of the Federation of Medical and Allied Societies met at the council room of the Medical Society of London on November 6 to consider the effect of Clause 11 of the Ministry of Health Bill. A series of resolutions was down for discussion, and Lord Knutsford, Captain Eliot, M.P., Colonel Fremantle, M.P., Sir Thomas Horder, M.D., Sir Henry Craik, Sir John Young, and others joined in the debate. With some small dissent it was resolved "that this conference of representatives of medical and allied organisations, whilst desirous of assisting the Minister of Health in his efforts to increase the efficiency of the health services of the country, regrets that it has been found necessary to introduce health legislation involving important principles in a Miscellaneous Provisions Bill." A further resolution expressed a demand that the Ministry should consult collectively the medical, dental, pharmaceutical, nursing, and midwives' professions, and yet another "that this conference suggests that the British voluntary hospitals' system be supplemented by State aid, but that the system of patients' contributions toward the expense of their maintenance in voluntary hospitals with Poor-law infirmaries should be given a fair trial." The meaning of the last resolution is a little obscure, but the sense of the meeting favoured a linking-up of voluntary hospitals with Poor-law infirmaries. The resolutions passed at the meeting, we are interested to note, were very much on the lines of our leading article of last week. The sensation of the afternoon was caused by Viscount Knutsford in announcing that in his belief the voluntary system has come to an end, and that the London Hospital will close on January 1. With the financial aspect of this situation we deal on another page.

### LORD KNUTSFORD'S HOSPITAL SCHEME.

Viscount Knutsford stated at the Federated Societies' meeting that his ideal scheme would be for the London County Council to make itself the health authority of London, and that its hospital system should embrace central hospitals and outlying hospitals. All the greater hospitals should remain as consultative centres. Poor-law infirmaries, with really good resident staffs, should be municipal hospitals and the outlying hospitals of the scheme. The schools would be at the centres, and cases which the outlying hospitals would not be able to attend to should be sent to them. The sources of income would be partly rate-supplied and partly voluntary. It would be a mistake to think that the voluntary hospitals were meeting the wants of London to-day, and those interested in them had better face the fact frankly. Why were people so frightened when they heard of State aid and State management? Hospitals were already getting money from municipal bodies, who contributed largely for the treatment of venereal, maternity cases, and so on, but did not interfere with the hospitals. Lord Knutsford has since greatly modified his statement as to the closing of the London Hospital. He now says that it may be possible that some of the work, perhaps on the scientific side, will have to be discontinued.

### HOSPITALS AND NATIONAL HEALTH INSURANCE.

Of suggestions for raising funds for hospitals there are many, and one of the latest comes from Mr. R. J. Coles, the Secretary of King's College Hospital. He thinks it might be arranged that an extra weekly 2d. could be paid in respect of insured persons under the National Health Insurance Scheme, this amount to be collected through the purchase and use of stamps and distributed to hospitals by the Ministry of Health. For London alone, Mr. Cole calculates, the yield would be £1,000,000 a year, and this sum would meet the estimated deficit and leave something over for long-needed renewals and extensions. At the eighth annual meeting of the National Conference of Industrial Assurance Approved Societies, held on Monday, the President, Mr. A. C. Thompson, stated that it was anticipated that the societies would show a substantial surplus as a result of their first valuation, now being proceeded with by the Government actuary. The question would arise as to the best means of applying this to the greatest advantage of the members. The provision of treatment in hospital might become a matter of extreme value to the insured person in the very near future. If, by way of additional benefit, Societies were able to provide for their members' free hospital treatment, they would in many cases confer a benefit far outweighing the value of increased cash payment. Mr. Thompson's plan does not suggest an entirely new source of income, it means that the approved societies would meet patients' payments instead of the patients themselves. It would, however, simplify collection and probably in the aggregate bring more cash into the hospitals' exchequer.

### THANK GOODNESS FOR AN OPTIMIST!

We like Mr. Arthur S. Morley, who, while Lord Knutsford tells us that the voluntary hospital system is tottering to its fall, joins optimistically in the correspondence in the *British Medical Journal* on "A Solution to the Hospital Problem." Is he the Mr. Arthur Morley of the Royal National Orthopædic Hospital? If so, our liking dates from before this week. Whichever Mr. Morley he is, he has some ideas; it is true that they are ideas which require extensive education of the British public, but they are none the less practical for that. His key idea is that people must be taught to believe that it is "good form" to give to hospitals. Everybody, he says, tips a waiter, a taxi-driver, and other people, because it is well recognised that it is "bad form" not to do so. We venture to interject the reflection that in these circumstances good form is also a qualified nuisance, but that does not destroy the argument. Tip, we do. By a process of advertising Mr. Morley suggests it might be made a question of "good form" to pay 5 per cent. or 10 per cent. to one of the hospital funds on restaurant and hotel bills, the money being collected and handed over to the proprietors of the establishment in question. Customers are asked to buy hospital stamps and have them affixed to the bill at the moment of settling. The system, it is suggested, could be extended to stockbroking transactions, to luxury buying, etc. Another good suggestion is the family collecting-box, which should stand in the hall of every family who will fall in with the prevailing fashion of giving to hospitals and into which should go one penny per week for each person resident in the house. We like Mr. Morley's enterprising ideas and are glad to learn that they have been in the hands of the Red Cross Society and are receiving careful consideration.

### NEW SOURCES OF REVENUE.

Secretaries of hospitals, who, of course, are always on the look-out for new sources of revenue, should not neglect pressing the claims of their institutions on the organisers of shows and exhibitions. A word in due season may do much toward directing into hospital exchequers money which may otherwise flow into less needy, not to say less worthy, coffers. A case in point is provided by the Tredegar Horse Show, a South Wales organisation, which has found itself with a surplus of £600 after its expenses have been paid. One is glad to note that of this total £300 has been voted by the Committee to the Tredegar Cottage Hospital and £100 to the Nurses' Home.

### THE DUKE OF YORK AT THE HOMŒOPATHIC.

The recent visit of the Duke of York, attended by Wing-Commander Louis Greig, to the London Homœopathic Hospital was much enjoyed by both staff and patients. He was received by the President, Major-General Lord Cheylesmore, the Secretary, Major Attwood, and the Matron. At his own request the sisters were presented to him, also Dr. John Weir, Dr. Edwin Neatby, Senior Gynæcological Physician, Mr. James Johnstone, Senior Surgeon, and Dr. Giles F. Goldsbrough. In the Durning Ward the Duke spent some time talking to Private Bullock, who served with the Prince of Wales at Bethune, also with other military patients. When his presence was realised by the out-patients they raised a cheer and were more than rewarded by the Duke shaking hands with some of their number. He expressed to the Board his hope that the hospital, which like the majority of voluntary institutions is in serious financial difficulties, would meet with the support it deserves.

### WAR MEMORIAL IN A POOR-LAW INFIRMARY.

The first war memorial erected in the precincts of a Poor-law infirmary to the memory of the men who have died there whilst the institution was utilised by the Army Council as a hospital for wounded soldiers was unveiled on Monday afternoon at the Southwark Hospital. In three years and five months 12,522 wounded and sick soldiers came to the hospital, and of these 199 made the supreme sacrifice. It was only fitting, therefore, that a memorial should be erected to the memory of the non-commissioned officers and men who passed away. The memorial is elegant, yet simplicity exemplified. It is of best Portland stone, twelve feet high, fixed on concrete foundations in the grounds immediately in front of the main entrance. A slender column is surmounted by a plain Celtic cross, the basement stone bearing the following inscription: "To the memory of the non-commissioned officers and men of the armies of the British Empire who died for freedom and honour during the Great War, 1915-19." Round the three remaining sides are inscribed the names of the non-commissioned officers and men.

### A CHAIR OF TUBERCULOSIS FOR WALES.

Mr. D. W. Evans, director of the Welsh National Memorial Association, announced at the recent meeting of the Council that Major David Davies, M.P., chairman, and his two sisters had presented the Association with £12,500 to found a Chair of Tuberculosis at the Welsh National Medical School, in memory of their grandfather, the late David Davies of Llandinam. The first professor, who will be nominated by the Council of the Association, will also be chief medical officer to the Welsh National Memorial Association. This will link the medical work of the Association to the National Medical School. The appointment will be made at an early date. The new professorship will be the second of its kind in the country. The first Chair of Tuberculosis was founded at the University of Edinburgh.

### WELSH TUBERCULOSIS QUESTIONS.

The numerous activities of the Welsh National Memorial Association show how difficult it is to make steady progress with the work. There are 144 vacant beds in the sanatoria and no waiting-lists. But before cases outside Wales can be considered, the very large waiting list of cases of surgical tuberculosis present a difficulty. It is hoped to make much greater use of the North Wales Sanatoria for these cases. The Association has bought a house and eighty-six acres at Sealyham, where a sanatorium for Pembrokeshire will be provided almost at once. The new institution at Pontypool, where courses of training are given in market-gardening and carpentry to ex-Service men, is not being well patronised. It seems that the scheme is generally known, but that insufficient provision is made for the families of the men during their absence. The Ministry of Health is to be asked to receive a deputation in the hope of removing this difficulty. This is a humble instance of the fact that the economic difficulty is the root of the consumptive problem. That is why its prevention is so largely beyond the power of medical men and science.

### A DISASTROUS MISUNDERSTANDING.

It is well known that testators do not always achieve the ends for which their wills were intended, and the late Mr. Robert Ainslie would have been much distressed if he had realised that the first effect of his will would be to embarrass the Edinburgh Royal Infirmary. It having gone abroad that by his will the institution is to receive a very large addition to its capital, the present efforts to help its finances have been largely unsupported. Mr. W. S. Caw, the treasurer and clerk, has, therefore, had to explain the true position. When Mr. Ainslie died in 1900 he left his fortune to accumulate for fifteen years with instructions to his trustees to provide and endow a convalescent home for the patients of Edinburgh Royal Infirmary. When the period expired in 1916 the war prevented immediate action, but the trustees have lately submitted a draft deed to the Court, which, if approved, will place the funds in the hands of a separate body of trustees. When the new convalescent home is eventually opened, the Royal Infirmary will have a very valuable adjunct. But, meantime, the infirmary itself has to be kept going, and will in no case benefit financially either now or later from the will. When we mentioned the matter some weeks ago we made this point quite clear. It is most unfortunate, therefore, that the will has interfered, through public misapprehension, with efforts essentially needed to keep the infirmary going.

### SMALLPOX AGAIN.

We understand that some sixty cases of smallpox are now under treatment in the neighbourhood of Middleton, near Manchester. We note that the local authorities are hastening ahead with isolation hospital hut erection in case of further spread of the disease, which, in a dense mill and workshop, is a wise precaution, in view of the fact that local hospital accommodation is already overtaxed. Re-vaccination measures are, however, probably the most urgent necessity in this part of the country, where the indifference of the population to this obvious means of defence is notorious. The utmost is being done in this direction, and no campaign could be more deserving of success.

### A SHEAF OF STATISTICS.

From Ancoats Hospital, Manchester, we have received an elaborate statistical report of the work of the hospital, medical and financial, for the nine months of this year ending September 30, and compared with the like period in 1918 and 1919. This report reflects great credit on the industry of its compiler, and is especially instructive, as it illustrates the incidence of the high cost of maintenance on a hospital in a great city. The volume of work in 1920 is practically the same as in 1919. The income has increased from £13,240 to £16,203 (annual subscriptions, as one item, from £3,055 to £4,232). The expenditure has increased from £16,127 to £21,261. £2,250 has been received for endowment and £22,466 for the Nurses' Home Building Fund Account. The consumption of meat has risen from 14,443 to 17,186 lb., but that of butter, bacon, bread, and milk has decreased. Statistics are provided in respect of the use of water, gas, electric light, coal, and the chief articles used under the heading of surgery and dispensary. The whole report is an indication of efficient management.

### HOSPITAL DIFFERENCES AT BIRMINGHAM.

The Committee of the Birmingham Children's Hospital (King Edward VII. Memorial) has been criticising the action of the General Hospital in declining to allow the League, recently founded to raise funds for the hospital, to work for the benefit of the city hospitals generally. The Children's Hospital Committee urges that "the scheme is so far-reaching, aiming to secure regular and permanent contributions from the workers and others, that it would be likely to damage the prospects of other hospitals when making their appeals. The Committee believes that if the League had been made comprehensive, it might eventually do for Birmingham that which King Edward's Hospital Fund has done for London." Our readers may remember that for a long time abortive attempts have been made to found a fund like King Edward's Hospital Fund for Birmingham and the Midlands. The protest of the Children's Hospital Committee is the latest instance of this unsuccessful desire, and without forgetting the needs of the General Hospital, we wish that the large issue had not been again allowed to fail, for clearly were it realised the benefit to the Birmingham hospitals would be greater than the success of the General Hospital's own League can be.

### ANXIOUS DAYS AT EXETER.

With the heavy expenses, amounting to £35,000, on the new buildings at the Devon and Exeter Hospital, and the cost of administration rapidly increasing, the Committee's anxieties during the coming year are unusually great. The Building Fund amounts to £23,000 at present, and £3,000 a year are expected from patients' payments, but unless the debt can be paid investments will have to be sold, and the hospital will lose £1,500 from this source. Five promises of £100 have been received in response to a preceding offer, conditional upon £2,000 being raised by similar sums. Gifts in kind, from an egg week and a potato week, have saved the hospital several hundred pounds. But additional income is badly needed. In connection with the new buildings, an enlargement of the pathological department has been approved. The conditions are such at present that the staff has run risks of blood-poisoning, because of the unfitness of the quarters for this work. The cost of building is increased because of the restrictions imposed on the workmen; only six are allowed where sixteen could easily be employed. A representative of the local Trades and Labour Council has suggested that the Governors should not bind themselves in the future to one architect, because by so doing they limit themselves to builders who will contract for the work under him. Meantime, however, the work is delayed and made expensive, because of the restrictions imposed.

### WARD DECORATION AT ROCHESTER.

Three interesting war memorials have been dedicated at St. Bartholomew's Hospital, Rochester. An endowed cot and a tablet in St. Mark's Ward is the gift of the officers and men of Chatham Dockyard. The Children's Ward has been presented with a set of tile-pictures by Mr. and Mrs. H. Hales, of Paradise Farm, Hartlip, and part of the scheme of decoration by St. Nicholas' Guild, Rochester. The war fund for the dependants of men who joined the Forces from the dockyard was continued to provide the above war memorial, and, further, a weekly collection in aid of local hospitals is now being organised in the dockyard. The ten tile-pictures depict fairy tales and Bible stories.

### WEEKLY CONTRIBUTIONS IN BOURNEMOUTH.

Another instance of the successful start of weekly contributions in a district previously almost unorganised in this respect occurs at Bournemouth. The hospital committee there has begun to arrange small contributions from employees in every place of business, large or small. The contribution is either one penny per week, or even threepence, or a contribution at the rate of ½d. for every ten shillings earned, or, in other cases, a lump sum monthly or quarterly. So far some hundred and thirty firms and their branches have adopted the plan, which of its nature is infectious. If it becomes the rule, then the proposal on the eve of accomplishment to close a ward at the Boscombe Hospital and the branch in Lowther Road may be avoided. But the point is that if the plan can be made successful in Bournemouth it can be successful anywhere, even in London, the recalcitrant area so far as the adoption of weekly contributions is concerned. At all events the system is gradually creeping from industrial to residential areas, and our persistent advocacy cannot help delighting in every new instance of its adoption.

### THIS WEEK'S DRUG MARKET.

There is no improvement in the demand for drugs, and the downward tendency of prices is still strongly in evidence. Makers of potassium citrate, sodium citrate, and iron and ammonium citrate have reduced their quotations in consequence of the decrease in cost of citric acid. Coca butter is offered at rather lower rates. Menthol and Japanese peppermint oil are unchanged. Quinine is available at rather lower rates. Santonin is cheaper and supplies are more plentiful. Potassium bromide is again lower. Eucalyptus oil is in rather better demand again, and prices are firmly maintained. Ergot of rye is available at rather lower prices. Formaldehyde is more plentiful and rather cheaper.

